# Relationship between Electrically Evoked Compound Action Potential Thresholds and Auditory, Language, and Speech Progress after Cochlear Implant Surgery 

**Published:** 2018-07

**Authors:** Masoud Motasaddi Zarandy, Navid Nourizadeh, Farzad Mobedshahi, Sadegh Jafarzadeh

**Affiliations:** 1 *Department of Ear, Nose and Throat,* * Faculty of Medicine,* * Tehran University of Medical Sciences, Tehran, Iran.*; 2 *Sinus and Surgical Endoscopic Research Center, Mashhad University of Medical Sciences, Mashhad, Iran.*; 3 *Amir Alam Hospital, Tehran University of Medical Sciences, Tehran, Iran.*; 4 *Department of Audiology, School of Paramedical Sciences, Mashhad University of Medical Sciences, Mashhad, Iran.*

**Keywords:** Cochlear implant, Electrically evoked compound action potential, Neural response telemetry, Newsha

## Abstract

**Introduction:**

Electrically evoked compound action potential (ECAP) is an objective auditory response that can be used in the programing of cochlear implants. The aims of this study were to monitor ECAP thresholds and auditory, language and speech progress for 6 months after cochlear implant surgery and to evaluate any relationship between them.

**Materials and Methods:**

Ten children with a mean age of 4.2 (±0.6) years and bilateral congenital and profound sensorineural hearing loss underwent cochlear implant surgery and post-operation auditory and speech training. The auditory, language, and speech abilities (Newsha level) and ECAP thresholds (for apical, medial and basal region of cochlea) were evaluated 1, 3 and 6 months after surgery.

**Results:**

ECAP threshold showed no significant improvement in any of the evaluated areas in the 6 months after surgery (P>0.05); however, the Newsha level improved for all patients (P=0.00).

**Conclusion:**

There was no relationship between ECAP thresholds and auditory, language, and speech abilities (Newsha level) in the first 6 months after surgery. ECAP thresholds may be a poor indicator of improvement in auditory, language, and speech abilities, and depend on many factors.

## Introduction

Electrically evoked compound action potential (ECAP) is an objective auditory response that is achieved by the neural response telemetry (NRT) method and may be used in the programing of cochlear implants (1,2). This response shows the function of the cochlear implant and auditory nerve (3), and can be tested during and after surgery. The thresholds decrease after surgery (1,4), due to electrode interactions with the surrounding tissue. However, there is some disagreement about further improvement of ECAP thresholds over the subsequent months after surgery. This may be important, because improvement of ECAP thresholds show better synchronization in the auditory nerve and may relate to patients’ auditory, language, and speech progress (5). Also, new thresholds may be used in the reprogramming of the device.

In this study, ECAP thresholds and scores on the Newsha developmental scale were recorded in the 6 months after surgery. The Newsha developmental scale is a Persian scale that evaluates the auditory, language, speech, and cognition ability of infants and children up to 6 years of age (6), and has good validity and reliability (7). The aims of this study were to monitor ECAP thresholds and investigate any relationship between ECAP thresholds and Newsha levels in the 6-month period after cochlear implant surgery.

## Materials and Methods

*Participants*

Ten children, aged 3 to 5 years, participated in this study. All children had bilateral congenital and profound sensorineural hearing loss. The children were selected randomly and had no other abnormalities such as auditory neuropathy, mental retardation, or autism. Inclusion criteria were congenital profound sensorineural hearing loss in both ears, history of bilateral hearing aid use for at least 6 months, unsatisfactory improvement in auditory, language, and speech abilities despite rehabilitation before surgery, and normal radiologic evaluations for cochlear implant surgery. Auditory sensitivity was evaluated using behavioral audiometry (play audiometry, tympanometry, and acoustic reflex) and physiologic assessment including otoacoustic emissions, auditory brainstem responses, and auditory steady state response.

The procedures were explained to the children and their parents, and all provided informed consent to participate in this study. All evaluations were free of charge. This study was approved by the ethics committee of Tehran University of Medical Sciences.

*Procedure*

Participants underwent cochlear implant surgery and received Freedom C5 (Nucleus) for one ear. All surgeries were performed under general anesthesia using mastoidectomy and round window insertion of electrodes, as this method shows better placement of electrodes than cochleostomy (8).

All surgeries were performed by a single person.Participants underwent routine auditory and speech training after surgery, including auditory verbal therapy. The ECAP thresholds and Newsha level were evaluated 1, 3, and 6 months after surgery. ECAP thresholds were recorded by NRT for the apical (electrode number: 22), medial (electrode number: 11), and basal region (electrode number: 2) of the cochlea, and the Newsha level was determined by a trained audiologist and speech and language pathologist.

*Data analysis*

Data were analyzed with SPSS 19 software using descriptive analysis and analysis of variance (ANOVA) for ECAP thresholds and generalized estimating equations for the Newsha level.

## Results

The mean (standard deviation) age of participants was 4.2 (0.6) years, and seven out of 10 were female. Surgeries were performed on the right side in all patients, and they attended regular rehabilitation sessions after surgery as scheduled.

Table 1 shows the results of ECAP responses and Table 2 shows Newsha levels 1, 3, and 6 months after surgery. These results show that the ECAP responses had no significant improvement over 6 months (P>0.05), but the Newsha level improved significantly(P=0.00).

**Table 1:** Mean ECAP thresholds (current level) 1, 3, and 6 months after surgery

	**ECAP responses**		
	**Apical**	**Medial**	**Basal**
1 month after surgery	144.60 (31.54)	177.20 (17.76)	178.70 (25.87)
3 months after surgery	146.00 (25.94)	173.30 (14.61)	177.20 (18.41)
6 months after surgery	147.70 (25.11)	174.00 (12.06)	180.40 (11.75)

**Table 2:** Frequency (number of patients) in each Newsha levels 1, 3, and 6 months after surgery

	**Newsha level**										
	**1**	**2**	**3**	**4**	**5**	**6**	**7**	**8**	**9**	**10**	**11**
1 month after surgery	6		1		2	1					
3 months after surgery					3	3	1	2	1		
6 months after surgery							2	5	1	1	1

## Discussion

ECAP thresholds showed no significant improvement in any of the evaluated areas in the 6 months after surgery, but the Newsha level improved for all participants in the same period. This finding is similar to previous studies that found no change up to 12 months after surgery or only a small reversible change in ECAP threshold, but contrast with other studies that showed an improvement or increase of ECAP thresholds (1-14). 

Studies that showed improvement of ECAP thresholds also have some inconsistencies; some showed improvement only in the basal area (11), or the basal and apical areas but not the medial area (13), or the apical and medial areas of the cochlea only in first 3 months after surgery (12).

The ECAP improvement may represent better neural connection and synchronization in the peripheral auditory system (5); however, it does not necessarily lead to better processing in the auditory system (15). Furthermore, ECAP thresholds depend on many pre- and post-surgery factors including the status of the nervous system before surgery, the type and amount of neural stimulation received, pathologic conditions of the inner ear such as stenosis of the cochlear nerve canal, electrode type, position, insertion method, device type, patient age (younger than 18 months), and presence of neural abnormalities such as auditory neuropathy (4,8,11,16-20). However, they do not relate to the insertion depth of the electrodes or the size of the cochlea (17).

On the other hand, improvement in Newsha levels show better auditory and speech abilities and good neural stimulation through cochlear implantation. Therefore, it may be inferred that improvements of ECAP threshold depend on many factors, and some patients (including our participants) may show no improvement.

It has been reported that ECAP thresholds do not have a good correlation with behavioral thresholds (21), and the results of this study and related studies may suggest that ECAP is a poor indicator of patient improvements in auditory, language, and speech ability. One weakness of the current study is that it used only one type of cochlear implant device. The results may be different for other types of device, and our suggestion therefore is for further studies to be conducted using different devices.

## Conclusion

There was no relationship between ECAP thresholds and auditory, language, and speech abilities (Newsha level) in the first 6 months after surgery. ECAP thresholds may be a poor indicator of improvement in auditory, language, and speech abilities and depend on many factors.
